# Prevalence of risk stomach in laboring women allowed to unrestrictive oral intake: a comparative cross-sectional study

**DOI:** 10.1186/s12871-022-01582-z

**Published:** 2022-02-07

**Authors:** Xiang-Yang Chang, Li-Zhong Wang, Feng Xia, Yin-Fa Zhang

**Affiliations:** Department of Anesthesiology, Jiaxing Maternity and Children Health Care Hospital, Affiliated Women and Children Hospital, Jiaxing University, Jiaxing, 314000 Zhejiang China

**Keywords:** Gastric ultrasound, Gastric volume, Labor, obstetric, Obstetric anesthesia, Pulmonary aspiration

## Abstract

**Background:**

Although restricting food intake during labor is recommended by guidelines, intrapartum starvation has not been popular in some regions. We conducted this comparative cross-sectional study to determine the prevalence of risk stomach in non-fasted laboring women compared with fasted non-laboring women using gastric ultrasound.

**Methods:**

Ultrasound examination of the antrum was performed in 50 term fasted non-laboring women before elective cesarean delivery and 50 laboring women allowed to eat and drink during active labor. Examinations consisted of the qualitative (antral grades, 0–3) and quantitative evaluation (antral cross-sectional area and calculated gastric volume) in the supine and right lateral decubitus (RLD) position. A risk stomach was defined as an antral grade ≥ 2 or grade 1 with gastric volume ≥ 1.5 ml· kg^− 1^.

**Results:**

No non-laboring women had grade ≥ 2, while 34 (68%) laboring women had grade ≥ 2. Nine (18%) non-laboring and 40 (80%) laboring women presented risk stomach (*P* < 0.001) (risk ratio: 4.4, 95% CI 2.4–8.2). Compared with non-laboring women, laboring women had larger antral area at “empty” stomach (grade 0) (437 mm^2^ vs.350 mm^2^ in supine, 571 mm^2^ vs.480 mm^2^ in RLD, *P* < 0.05) and cut-off values of antral area to discriminate a risk stomach (510 mm^2^ vs. 453 mm^2^ in supine, 670 mm^2^ vs. 605 mm^2^ in RLD).

**Conclusions:**

This study confirms a higher prevalence of risk stomach presents in laboring women under a liberal eating policy, gastric ultrasound is therefore useful for this risk population if general anesthesia is required unexpectedly.

## Background

Although regional anesthesia is preferentially used for most obstetric procedures in obstetric anesthetic practice, [1] there are still some situations in which general anesthesia may be necessary, especially in case of emergency cesarean delivery during labor. To reduce the risk of pulmonary aspiration, guidelines recommend a restriction of oral intake to clear liquids during labor for low risk patients, with further restriction for those at increased risk for aspiration [1, 2]. Nevertheless, restricting food intake during labor is controversial [3–5]. Moreover, oral intake management measures during labor vary depending on different cultures and regions. In china, for example, childbirth is traditionally considered as a process in which the mother continuously consumes energy, and supplementing food is beneficial. Therefore, a relatively liberal oral intake policy on the labor ward is adopted in about 77% hospitals in China, [6] which may significantly increase the risk of aspiration when general anesthesia is required unexpectedly.

Gastric ultrasound is a valuable tool for the assessment of gastric contents and volume in obstetric anesthesia, [7] including non-laboring [8–14] and laboring women [15–19]. However, most studies on laboring women have been studied in those completely fasted or only allowed to drink clear liquids. There is insufficient information regarding the impact of such a liberal eating policy on the gastric contents in laboring women. Thus, we conducted this comparative cross-sectional study to determine the prevalence of risk stomach in term non-fasted laboring women compared with fasted non-laboring women using ultrasound examination of the antrum. In addition, we also estimated the ability of antral cross-sectional area (CSA) to identify a risk stomach in this setting.

## Methods

This comparative cross-sectional study was approved by the local ethics committee at Jiaxing Maternity and Children Health Care Hospital (approval # 2020-F-1) and registered with the Chinese Clinical Trial Registry (http://www.chictr.org.cn/showproj.aspx?proj=53653) (ChiCT2000033900). The date of registration approval was on 16 June 2020, and the study was conducted from 1 July to 23 October 2020. Written informed consent was obtained from all enrolled subjects. The study was designed and conducted in accordance with the Strengthening the Reporting of Observational Studies in Epidemiology (STROBE) guidelines [20].

Non-laboring cohort consisted of women scheduled for elective cesarean delivery under neuraxial anesthesia, while laboring cohort was those for vaginal delivery in active labor (from cervical dilation ≥3 cm until full cervical dilatation) with or without epidural analgesia. The inclusion criteria were age ≥ 18 years, term (> 37 weeks’ gestation), a singleton fetus with cephalic presentation, and ASA physical status I to II. Exclusion criteria were multiple gestations, body mass index (BMI) ≥35 kg·m^− 2^, a history of gastric or oesophageal disease or surgery, gastroesophageal reflux disease, diabetes mellitus and refusal to participate in the study. Non-laboring women followed institutional fasting guideline (a minimum of 2 h for clear liquids, 6 h for a light meal, and 8 h for a full meal). No premedication was given before cesarean delivery. Laboring women were allowed to eat easily digestible foods and drink liquids as they wish throughout the course of labor.

Women were consecutively recruited during the study period according to the ultrasound operator availability who had experience of at least 50 gastric ultrasound examinations in pregnant women. Ultrasound examination was performed immediately prior to neuraxial anesthesia in operation room, or at varying times in active labor regardless of oral intake in labor ward. A standardized scanning technique was used with a portable ultrasound system and a 2–5 MHz curved array transducer (Navi ultrasound system, Shenzhen Wisonic Medical Technology Co., Ltd., Shenzhen, China). The gastric antrum was imaged in a sagittal plane in the epigastrium between the left lobe of the liver and the aorta. A detailed description of the technique and ultrasound characteristics of antrum contents has been previously described in the literatures [7, 21–23]. Women were first scanned in the supine position, followed by the right lateral decubitus (RLD) position, both with the head of the bed elevated to 45^°^. Examinations consisted of a qualitative and quantitative evaluation. The qualitative assessment determined the nature of gastric contents, including empty (flat antrum with juxtaposed anterior and posterior walls), fluid (distended antrum with thin walls and hypoechoic content) or thick fluid/solid food (distended antrum with content of mixed echogenicity). Based on the qualitative findings, women were classified into grade 0 (no content visible in either position), grade 1 (clear fluid content visible in RLD position only) and grade 2 (clear fluid content visible in both positions), according to the Perlas grading system [23]. For the present study, we further defined an additional grade 3 as the visualization of thick fluid/solid content in the antrum, whatever the position [16].

The quantitative assessment measured the CSA of the entire antral wall between peristaltic contractions, using the free-tracing caliper of the ultrasound system. The average of three consecutive measurements in each position was calculated for gastric volume estimation, according to the following mathematical formula validated in the late pregnant women using magnetic resonance imaging (MRI) as a reference standard, with adjusted *R*^2^ = 0.76: gastric volume (ml) = 0.18× supine-CSA (mm^2^) + 0.11 × RLD-CSA (mm^2^) − 62.4 [12]. This model applies to any gastric contents, rather to clear fluids only. The gastric volume per weight was calculated by using the current body weight. For this study, a risk stomach was defined as antral grade ≥ 2, or grade 1 with a calculated gastric fluid volume ≥ 1.5 ml· kg^− 1^, whereas a low-risk stomach was defined as grade 0, or grade 1 with a calculated gastric volume < 1.5 ml · kg^− 1^, as suggested in the recent literatures [7, 10, 21].

Women characteristics (age, gravity, parity, gestational age, weight) and the time intervals between last solid and liquid ingestion and ultrasound examination were recorded. In laboring women, the presence of epidural analgesia, the pain intensity assessed by using a 0–10 numerical verbal rating scale (0 = no pain and 10 = worst pain imaginable) and degree of cervical dilatation at the ultrasound examination were also noted. In addition, any failure to visualize the antrum was documented and excluded from the analysis.

### Statistical analysis

The primary outcome was the incidence of risk stomach in both cohorts. Secondary outcome was the antral CSA to discriminate a risk stomach. Continuous variables were expressed as mean (standard deviation, SD) or median (interquartile range). The Shapiro-Wilk test was used to test for normal distribution. Variables that were normally distributed were analyzed using the independent *t* test, while those not normally distributed were analyzed using Mann-Whitney *U* or Kruskal-Wallis test, as appropriate. Categorical variables were expressed as number (%) and analyzed using *x*^2^ or Fisher exact tests. Spearman rank correlation coefficient was estimated to determine the relationship between the duration of fasting and antral CSA. To assess the ability of antral CSA to identify a risk stomach, a receiver-operating characteristic (ROC) curve was generated and the area under the ROC curve (AUC) with 95% confidence interval (CI) was calculated. The cut-off value was determined based on the Youden index.

Based on a 9% incidence of risk stomach in fasted non-laboring pregnant women reported in a previous study, [10] and an assumption of at least 50% of non-fasted laboring women having risk stomachs, a minimum sample size of 23 women was required in each cohort to test the hypothesis with a significance level of 0.05 and power of 0.9. We decided to recruit 50 women in each cohort to make sample size more powerful. Data were analyzed using SPSS version 19.0 for Windows (IBM Corp., Armonk, NY, USA). A two-sided *P* < 0.05 was considered as statistically significant.

## Results

**Fig. 1:**
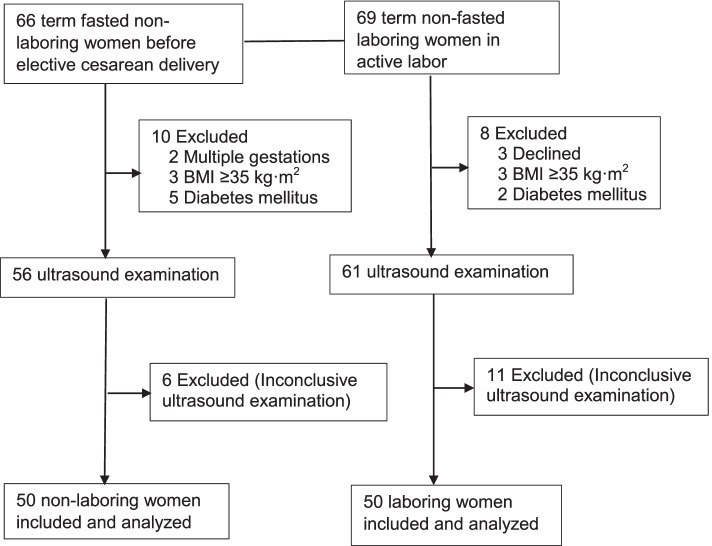
Flow diagram

Sixty-six non-laboring and 69 laboring women were assessed for eligibility. Eighteen women were excluded due to the exclusion criteria. Another 17 women were excluded because of inconclusive ultrasound examinations in one or both positions; of which, 6 occurred in non-laboring women, 11 occurred in laboring women. This corresponded to a rate of inconclusive examinations of 10.7% in non-laboring and 18.0% in laboring women, respectively. The 100 remaining women (50 in each cohort) were included in the final analysis (Fig. 1). All non-laboring women complied with the fasting guideline, while the median time interval from the last oral intake to ultrasound examination in laboring women was 1.0 h (range 0.2 to 5.5 h) for liquids and 3.0 h (range 0.2 to 8.0 h) for solids. Forty-five (95%) laboring women received epidural analgesia, the median pain intensity was 3 (range 1 to 6) and the cervical dilatation was 4 cm (range 3 to 8 cm) at the ultrasound examination.

Women baseline and ultrasound characteristics are summarized in Table 1. The antral CSA and the corresponding gastric volumes were significantly larger in laboring than in non-laboring women. There was no correlation between the fasting duration and antral CSA in non-laboring women; however, the fasting duration for solids but not for liquids, was significantly correlated to supine-CSA (*r* = − 0.334, *P* = 0.018) and RLD-CSA (*r* = − 0.327, *P* = 0.020) in laboring women. No non-laboring women had grade 2 or 3; in contrast, 34 (68%) laboring women presented grade 2 or 3 (*P* < 0.001). As expected, increased antral grades were associated with significantly larger antral CSA and thus greater calculated gastric volumes. When compared between the two cohorts, CSA in both positions for grade 0 were significantly larger in laboring than in non-laboring women (*P* < 0.05), but the differences for grade 1 did not reach statistical significance (*P* > 0.05) (Table 2).

**Table 1:** Baseline and ultrasound characteristics in non-laboring and laboring women

	Fasted non-laboring (*N* = 50)	Non-fasted laboring (N = 50)	*P-*value
Age (years)	30.0(3.7)	28.7(2.9)	0.060
BMI (kg·m^−2^)	26.1(3.1)	26.0(2.7)	0.883
Gestational age (weeks)	38.6(0.8)	39.0(1.3)	0.067
Gravidity, n (%)			< 0.001
1	9 (18)	13 (74)	
2	19 (38)	9 (18)	
≥3	22 (44)	10 (20)	
Parity, n (%)			< 0.001
0	13 (26)	37 (74)	
1	29 (58)	12 (24)	
≥2	8 (16)	1 (2)	
Fasting for solids (hours)	13.0 [11.7–15.0]	3.0 [0.8–4.0]	< 0.001
Fasting for liquids (hours)	11.5 [9.9–13.0]	1.0 [0.5–2.0]	< 0.001
Supine-CSA (mm^2^)	358 [328–437]	565 [450–843]	< 0.001
RLD-CSA (mm^2^)	557[462–646]	890 [661–1235]	< 0.001
Gastric volume (ml)	77.3 [59.5–98.6]	164.2 [108.9–251.1]	< 0.001
Gastric volume (ml· kg^−1^)	1.2 [0.9–1.5]	2.4 [1.6–3.6]	< 0.001
Antral grade, n (%)			< 0.001
Grade 0	33 (66)	8 (16)	
Grade 1	17 (34)	8 (16)	
Grade 2	0	7 (14)	
Grade 3	0	27 (54)	

Values are expressed as mean (standard deviation), numbers (percentages %) or median [interquartile range]

*Abbreviation: BMI* body mass index, *CSA* antral cross-sectional area, *RLD* right lateral decubitus

**Table 2:** Antral cross-sectional area, gastric volume and fasting duration according - the antral grades in non-laboring and laboring women

	Fasted non-laboring			Non-fasted laboring				
	Grade 0 (*N* = 33)	Grade 1(*N* = 17)	*P-*value	Grade 0 (N = 8)	Grade 1(*N* = 8)	Grade 2 (*N* = 7)	Grade 3 (*N* = 27)	*P*-value
Supine-CSA (mm^2^)	350 [307–407]	395 [349–472]	0.061	437 [386–494]^*^	446 [435–496]	841 [561–1542]	722 [555–930]	< 0.001
RLD-CSA (mm^2^)	480 [435–567]	653 [591–882]	< 0.001	571 [528–622] ^*^	741 [620–820]	1110 [917–2200]	1113 [790–1355]	< 0.001
Gastric volume (ml)	67.9 [50.9–78.4)]	102.4 [82.2–146.3]	< 0.001	85.3 [65.6–104.7]^*^	123.3 [98.0–136.2]	216.8 [170.0–505.2]	206.1 [160.2–289.0]	< 0.001
Gastric volume (ml· kg^−1^)	1.0 [0.8–1.2]	1.6 [1.2–2.1]	< 0.001	1.3 [1.0–1.5] ^*^	1.8 [1.3–2.1]	3.1 [2.4–8.0]	3.0 [2.3–4.3]	< 0.001
Fasting for solids (hours)	13.0 [12.0–14.5]	12.0 [10.5–15.0]	0.725	3.5 [1.9–5.1]	3.5 [1.1–4.8]	1.5 [0.7–4.0]	2.5 [0.5–3.5]	0.385
Fasting for liquids (hours)	12.0 [10.0–13.0]	11.0 [8.3–12.5]	0.680	1.0 [0.5–1.8]	1.0 [0.5–3.8]	0.7 [0.5–2.0]	1.0 [0.5–2.0]	0.886

Values are expressed as median [interquartile range]

*Abbreviation: CSA* antral cross-sectional area, *RLD* right lateral decubitus

^*^ Compared - non-laboring women with grade 0, *P* < 0.05

**Fig. 2:**
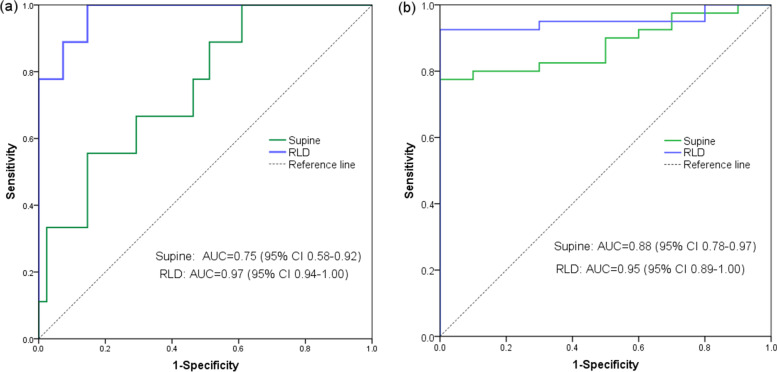
Receiver operating characteristic curves of antral cross-sectional area to discriminate a risk stomach in fasted non-laboring (**a**) and non-fasted laboring women (**b**). *P* < 0.05 for each curve. *Abbreviation: RLD* right lateral decubitus, *AUC* area under the receiver operating characteristic curve, *CI* confidence interval

There were 9 (18%) risk stomachs in non-laboring women and 40 (80%) risk stomachs in laboring women (*P* < 0.001) (risk ratio: 4.4, 95% CI 2.4 to 8.2). Notably, one (3%) of 33 non-laboring women with grade 0, while 2 (25%) of 8 laboring women with grade 0 had a calculated gastric volume ≥ 1.5 ml· kg^− 1^. According to the definition as described in the Methods, we still scored these women as low-risk stomachs. The AUC of CSA to discriminate a risk stomach in both cohorts are shown in Fig. 2. The RLD-CSA demonstrated a superior discriminatory performance than the supine-CSA. Based on the Youden method, the cut-off values of CSA to detect a risk stomach in non-laboring women were supine-CSA of 453 mm^2^, with a sensitivity (95% CI) of 56% (21 to 86%) and a specificity (95% CI) of 85% (71 to 94%), and RLD-CSA of 605 mm^2^, with a sensitivity of 100% (66 to 100%) and a specificity of 85% (71 to 94%); while in laboring women, the cut-off values were supine-CSA of 510 mm^2^, with a sensitivity of 77% (61 to 89%) and a specificity of 100% (69 to 100%), and RLD-CSA of 670 mm^2^, with a sensitivity of 92% (80 to 98%) and a specificity of 100% (69 to 100%).

## Discussion

Although gastric ultrasound has been studied in laboring women, [15–19] the present study differs from previous studies in terms of the studied subjects (allowed to eat and drink freely during labor) and study design (compared to fasted non-laboring women in one study). Our results confirm the impression that a higher incidence (80%) of risk stomach present in non-fasted laboring women. In addition, our study adds additional information to previous studies, i.e., laboring women have larger antral CSA at “empty” stomach and higher cut-off value of CSA to discriminate a risk stomach compared with non-laboring women.

Gastric ultrasound is a useful tool to assess gastric contents and volume at the bedside. In addition to the qualitative assessment, [23] several different algorithms have been proposed to predict gastric volume based on antral CSA in adult, non-pregnant subjects [23–25]. However, the potential changes in both the size and position of the antrum due to the gravid uterus at late pregnancy may alter the relationship between the antral CSA and gastric volume [14]. Recently two mathematical models for antral CSA have been described in pregnant women to predict gastric volumes [9, 12]. In the present study, we adopted the mathematical formula developed by Roukhomovsky et al. [12] mainly because this model used MRI rather than the ingested volume of fluid as a reference standard, and more importantly, it applied to any gastric contents rather to clear fluids only.

Although the definition of a full stomach that increases aspiration risk is somewhat controversial, recent literatures recommend that a full stomach may be defined as any solid or thick particulate content or grade 2, or grade 1 with gastric volume of ≥1.5 ml· kg^− 1^ under ultrasound examination [7, 10, 21]. Based on this criterion, the incidence (80%) of risk stomach in non-fasted laboring women in this study was significantly higher than the laboring women allowed to drink only in other studies (27 and 48%, respectively) [18, 19]. Moreover, the fasting time for solids, but not for liquids, was significantly associated with the antral CSA, suggesting that solid foods have a major contribution of risk stomach during labor. However, it should be noted that, despite the ultrasound showing “empty” stomach image (grade 0), one non-laboring woman and 2 laboring women have a predicted gastric volume exceeded 1.5 ml· kg^− 1^. Although we classified these women as low-risk stomachs, the interpretation of these women is difficult and controversial. As gastric ultrasound is likely most valuable when the pre-test probability of having a full stomach is in the order of 50%, [7] it can be therefore very useful to assess the risk of aspiration using bedside ultrasound in this population if emergent general anesthesia is required.

The qualitative finding of our fasted non-laboring women is similar to those previous studies, where the fasted women before elective cesarean delivery mostly had grade 0 or 1 antrum with no solid content visualized [8, 9, 11]. As regards the quantitative results, our results were close to those reported by Arzola et al. (Supine-CSA of 3.8 cm^2^ and RLD-CSA of 5.2 cm^2^), [9] but were higher than the values reported by other authors [10, 12]. However, Roukhomovsky et al. [12] performed ultrasound assessment on the pregnant women around 31 weeks, as opposed to the term women in our study; Putte et al. [10] stated merely that “the right lateral decubitus position”, not explicitly mentioned whether the head was high 45^°^. Also, various cut-off values of CSA to discriminate between “full” and “empty” stomach have been shown across different studies, with supine-CSA ranging from 320 to 505 mm^2^ [12, 15] and RLD-CSA from 870 to 1030 mm^2^ [8–10] in non-laboring women. In laboring women, the cut-off values of supine-CSA of 608 mm^2^ and RLD-CSA of 719 mm^2^ were proposed in one study [17]; conversely, a cut-off value of supine-CSA of 381 mm^2^ was reported in another study [16]. Different gastric volume estimation (mathematical models or ingested fluid volume), diagnostic criteria of a “full” stomach (combination of the qualitative and quantitative assessments or only gastric volume ≥ 0.8 or 1.5 ml· kg^− 1^), and the method for the calculation of cut-off value (ROC or the 95th percentile) may explain in part the disparity. In addition, as expected, the CSA in supine revealed a better discriminatory performance in both cohorts than the CSA in RLD position. This is consistent with the findings of previous studies that reported a more accurate assessment of gastric volume in the right lateral decubitus position, given that a greater proportion of the gastric content is displaced towards the more dependent antrum in this position compared to the supine position [22, 26].

We note that laboring women had larger antral CSA for grade 0 and higher cut-off value for identification of a risk stomach compared to non-laboring women. The exact reason for this difference is not clear, but it may be related to some factors during labor such as uterine contractions, pain, analgesia and hormonal changes, etc., that may affect the antral size, position and motility. Further studies of comparing laboring to non-laboring women are required to validate and clarify this finding. Our study also confirms that gastric ultrasound can be more difficult to perform on laboring than non-laboring pregnant women, [17, 19] as evidenced by the rate of inconclusive examinations. In addition to cephalad displacement of the stomach and the steep angle between the xiphoid process and abdomen, uterine contractions and particularly the presence of air after solid food ingestion can pose additional challenges to ultrasound visualization during labor.

Our study has several limitations. First, due to the relatively small sample, our results may not be accurate enough and should be considered with caution. Second, the gastric volume was estimated by using a mathematical model derived from non-laboring pregnant women rather than by assessing the actual gastric volume. This may impair the reliability of our results. However, the direct measurement of gastric volume was not feasible in this study. Third, due to the study design, the operator-blinded was actually not possible. Fourth, we did not record the type of the foods and liquids ingested and cannot comment on the impact of food type on the gastric contents. Finally, we did not include a cohort in which the laboring women were allowed to drink clear liquids only. Therefore, we cannot compare the laboring women under a liberal food intake to those under a less-restrictive oral intake in one study.

## Conclusion

Our study confirms a higher prevalence of risk stomach in laboring women under a liberal oral intake policy. Gastric ultrasound is therefore useful in this population if general anesthesia is required. Our findings also imply the potential differences in gastric ultrasound appearances between laboring and non-laboring women. Further studies are required to clarify the potential changes regarding the ultrasound characteristics of the antrum in laboring women.

## Data Availability

The datasets used and/or analysed during the current study available from the corresponding author on reasonable request.

## References

[1] Practice Guidelines for Obstetric Anesthesia (2016). An updated report by the American Society of Anesthesiologists Task Force on obstetric anesthesia and the Society for Obstetric Anesthesia and Perinatology. Anesthesiology.

[2] ACOG Committee Opinion No. 441: Oral intake during labor. Obstet Gynecol. 2009;114:714. 10.1097/AOG.0b013e3181ba0649. 10.1097/AOG.0b013e3181ba0649 19701066

[3] Singata M, Tranmer J, Gyte GM (2013). Restricting oral fluid and food intake during labour. Cochrane Database Syst Rev.

[4] Ciardulli A, Saccone G, Anastasio H, Berghella V (2017). Less-restrictive food intake during labor in low-risk singleton pregnancies: a systematic review and Meta-analysis. Obstet Gynecol.

[5] Phelps K, Deavers J, Seehusen DA, Stevermer JJ (2018). PURL: let low-risk moms eat during labor?. J Fam Pract.

[6] Huang CY, Luo BR, Hu J (2020). Investigation on the status of oral intake management measures during labor in China. Medicine (Baltimore).

[7] Perlas A, Arzola C, Van de Putte P (2018). Point-of-care gastric ultrasound and aspiration risk assessment: a narrative review. Can J Anaesth.

[8] Arzola C, Perlas A, Siddiqui NT, Carvalho JC (2015). Bedside gastric ultrasonography in term pregnant women before elective cesarean delivery: a prospective cohort study. Anesth Analg.

[9] Arzola C, Perlas A, Siddiqui NT, Downey K, Ye XY, Carvalho JCA (2018). Gastric ultrasound in the third trimester of pregnancy: a randomised controlled trial to develop a predictive model of volume assessment. Anaesthesia.

[10] Van de Putte P, Vernieuwe L, Perlas A (2019). Term pregnant patients have similar gastric volume to non-pregnant females: a single-Centre cohort study. Br J Anaesth.

[11] Popivanov P, Irwin R, Walsh M, Leonard M, Tan T (2020). Gastric emptying of carbohydrate drinks in term parturients before elective caesarean delivery: an observational study. Int J Obstet Anesth.

[12] Roukhomovsky M, Zieleskiewicz L, Diaz A, Guibaud L, Chaumoitre K, Desgranges FP (2018). AzuRea, CAR’Echo collaborative networks. Ultrasound examination of the antrum to predict gastric content volume in the third trimester of pregnancy as assessed by MRI: a prospective cohort study. Eur J Anaesthesiol.

[13] Arzola C, Cubillos J, Perlas A, Downey K, Carvalho JC (2014). Interrater reliability of qualitative ultrasound assessment of gastric content in the third trimester of pregnancy. Br J Anaesth.

[14] Rouget C, Chassard D, Bonnard C, Pop M, Desgranges FP, Bouvet L (2016). Changes in qualitative and quantitative ultrasound assessment of the gastric antrum before and after elective caesarean section in term pregnant women: a prospective cohort study. Anaesthesia.

[15] Bataille A, Rousset J, Marret E, Bonnet F (2014). Ultrasonographic evaluation of gastric content during labour under epidural analgesia: a prospective cohort study. Br J Anaesth.

[16] Jay L, Zieleskiewicz L, Desgranges FP, Cogniat B, Pop M, Boucher P (2017). AzuRea collaborative network. Determination of a cut-off value of antral area measured in the supine position for the fast diagnosis of an empty stomach in the parturient: a prospective cohort study. Eur J Anaesthesiol.

[17] Zieleskiewicz L, Boghossian MC, Delmas AC, Jay L, Bourgoin A, Carcopino X (2016). AzuRea and CAR'Echo collaborative networks. Ultrasonographic measurement of antral area for estimating gastric fluid volume in parturients. Br J Anaesth.

[18] Desgranges FP, Simonin M, Barnoud S, Zieleskiewicz L, Cercueil E, Erbacher J (2019). Prevalence and prediction of higher estimated gastric content in parturients at full cervical dilatation: a prospective cohort study. Acta Anaesthesiol Scand.

[19] Vial F, Hime N, Feugeas J, Thilly N, Guerci P, Bouaziz H (2017). Ultrasound assessment of gastric content in the immediate postpartum period: a prospective observational descriptive study. Acta Anaesthesiol Scand.

[20] von Elm E, Altman DG, Egger M, Pocock SJ, Gøtzsche PC, Vandenbroucke JP, Initiative STROBE (2014). The strengthening the reporting of observational studies in epidemiology (STROBE) statement: guidelines for reporting observational studies. Int J Surg.

[21] Perlas A, Van de Putte P, Van Houwe P, Chan VW (2016). I-AIM framework for point-of-care gastric ultrasound. Br J Anaesth.

[22] Van de Putte P, Perlas A (2014). Ultrasound assessment of gastric content and volume. Br J Anaesth.

[23] Perlas A, Davis L, Khan M, Mitsakakis N, Chan VW (2011). Gastric sonography in the fasted surgical patient: a prospective descriptive study. Anesth Analg.

[24] Perlas A, Mitsakakis N, Liu L, Cino M, Haldipur N, Davis L (2013). Validation of a mathematical model for ultrasound assessment of gastric volume by gastroscopic examination. Anesth Analg.

[25] Bouvet L, Mazoit JX, Chassard D, Allaouchiche B, Boselli E, Benhamou D (2011). Clinical assessment of the ultrasonographic measurement of antral area for estimating preoperative gastric content and volume. Anesthesiology.

[26] Perlas A, Chan VW, Lupu CM, Mitsakakis N, Hanbidge A (2009). Ultrasound assessment of gastric content and volume. Anesthesiology.

